# Relationship of anthropometric measures with Knee Osteoarthritis in diabetes mellitus

**DOI:** 10.12669/pjms.325.10425

**Published:** 2016

**Authors:** Saera Suhail Kidwai, Shaista Anwar Siddiqi, Lubna Nazir, Tahira Parveen Umer

**Affiliations:** 1 Saera Suhail Kidwai, MCPS, FCPS Associate Professor, United Medical and Dental College, Karachi, Pakistan; 2 Shaista Anwar Siddiqi, FCPS (Neurology). Strokologist; 3 Lubna Nazir, FCPS (Medicine), FCPS (Rheumatology). Liaquat National Hospital, Karachi, Pakistan; 4 Tahira Parveen Umer, FCPS (Medicine). Liaquat National Hospital, Karachi, Pakistan

**Keywords:** Knee Osteoarthritis (KOA), Diabetes Mellitus (DM), Body Mass Index (BMI), Waist circumference (WC)

## Abstract

**Objective:**

To determine the frequency of knee osteoarthritis in adult patients with Diabetes mellitus and its association with body mass index (BMI) in kg/m^2^ and waist circumference (WC).

**Methods:**

This is a cross-sectional comparative study at a tertiary hospital based in an industrial area of Karachi. Patient population comprised of all adult diabetic subjects. Clinical and demographic data was obtained with detailed musculoskeletal examination on all patients. BMI and WC were measured as kg/m^2^ and cm respectively. Data was analyzed on SPSS version 15.

**Results:**

A total of 413 subjects were recruited. Among them diabetic and non-diabetic subjects were 210 and 203 respectively. Mean age of diabetics was 50.7+-10.2 years as compared to non-diabetic subjects i.e. 49.5+-10.5 years. Proportion of male subjects was 72(34.3%) and 71(35.0) respectively in both groups. Mean duration of diabetes was 6.2 years. Frequency of knee osteoarthritis (OA) was found to be 52(24.8%) and 54(26.6%) in diabetic and non-diabetic respectively. Among the diabetic group 6 (18%) subjects with OA had normal BMI (18.5-22.9) whereas 4 (16%) were overweight (BMI 23-24.9) and 41(27.5%) were obese(BMI ≥25). Near 98% (n=51) of the diabetic patients with OA had high waist circumference. Females (n= 42, 31.1%) were more frequent than males (n=9, 16%) in the diabetic subjects with OA and a higher WC.

**Conclusion:**

Both diabetic and non diabetic group did not show any difference in the frequency of knee OA. However, frequency of knee OA showed a significant difference between overweight and obese category of BMI.WC appears as a strong predictor of knee osteoarthritis.

## INTRODUCTION

Osteoarthritis (OA), one of the most prevalent rheumatological conditions, is characterized by pain and limitations in daily activities caused by gradual deterioration and inflammation of the articular cartilages especially in the elderly.^1^,^2^ The etiology of knee osteoarthritis (KOA) is believed to be multi factorial including heredity, obesity, trauma, and physical workload. The impact of obesity on the risk for knee OA has been demonstrated in many studies. The effect however, may not only be attributed to the mechanical effect alone. Literature suggests that this effect may be mediated through a complex network of biochemical factors, including proteolytic enzymes, cytokines and other inflammatory mediators leading to cartilage erosion and synovial inflammation.^3^

The truncal and visceral adipocytes, in addition to releasing inflammatory mediators, also release the hormone *leptin*, abundantly seen in OA affected joints, which has been shown to have a direct destructive effect on joint chondrocytes.^4^-^6^ This argument is further supported by the relation between obesity and OA in non–weight-bearing joints, such as those in the hand.^7^-^9^

According to Niu et al, obese subjects; i.e a body mass index (BMI) of ≥30 kg/m^2^; have a significantly increased risk of incident kOA as compared to subjects with a normal BMI.^10^ Louise et al showed that the lifetime risk of symptomatic KOA (probability of a person developing symptomatic OA in at least one knee by age 85 years), was nearly 2 in 3 for obese persons at later ages as compared to 1 in 2 for overall study population.^11^

We have a very limited published data from the South Asian region which is of concern since the genetic heterogeneity and the variation in phenotypes are very important related to the incidence or prevalence of this debilitating disease which although not life threatening but the quality of life in those is affected beyond doubt. The immobility linked to the pain results in development of further obesity, lack of exercise and risk of Ischemic heart disease not to mention depression and mood disorders. We therefore, designed this study to assess the relationship of waist circumference and body mass index with the presence of KOA.

## METHODS

Data for this descriptive study was collected from the outpatient diabetic clinic as well as in patient of a tertiary care hospital located in an industrial area of Karachi. For Controls the attendants of the in patients were contacted after written informed consent approved by the ethical research committee. All subjects had to undergo a physicians led clinical and radiological examination if necessary and diagnosed as having osteoarthritis as declared by the ACR criteria of symptomatic knee OA which includes knee pain, joint space narrowing and morning stiffness of less than 30 minutes duration. Common variables assessed were age, sex, bodyweight in kilograms and height in centimeters, waist circumference in centimeters and duration of diabetes. To obtain a proxy measure of obesity, BMI profile was calculated from the available height (in meters) and weight (in kilograms) recorded using a standard laboratory scale and categorized as normal BMI with 18.5-22.9 kgs whereas, overweight with BMI of 23-24.9 kgs and obese as those with BMI of ≥25kgs. Waist circumference was taken as normal if ≤ 80cms in females and ≤90cms in males and high if ≥ 80 cms in females and ≥90 cms in males.^12^

SPSS version 16 was used for analysis. Descriptive statistics obtained were reported as mean and proportion with standard deviation (SD). Univariate association between diabetic and control groups was compared using Chi square test for categorical data and *t*-test for the continuous variables. P value of <.05 was considered as significant.

Logistic regression was used to examine the independent and combined influence of BMI and WC measures on knee osteoarthritis. For this analysis, the odds ratios (OR) were computed for each gender-specific standard deviation change in BMI and WC.

## RESULTS

In the one year recruitment period of this study a total of 413 subjects were included by convenient sampling, among them 210 were diabetic and 203 were non diabetics. Mean age was 50.7±10.2 years and 49.5±10.5 years in the diabetic and non diabetic subjects respectively Proportion of female subjects was 138(65.7%) and 132(65%) in diabetic and non diabetic groups respectively, whereas male subjects were 72(34.3%) and 71(35%) in both groups. Mean duration of diabetes was 6.2±5.7 years (Table-I). Frequency of knee osteoarthritis in diabetic and non diabetic cohorts was found to be 52(24.8%) and 54(26.6%) respectively (p = 0.66) (Table-II). Increasing age has shown increased frequency of OA in both groups (OR,I.03; 95% CI,1.01-1.06) Females are found to be twice more likely to suffer from OA than males both in diabetic and non diabetic groups (OR, 2.26; 95% CI, 1.29-3.6) (Table-III and IV) In the diabetic subjects, those in normal BMI category OA was present in 6 subjects(18%), whereas in overweight BMI category OA was present in 4 subjects(16%), however obese group has the highest risk of symptomatic knee osteoarthritis i.e. 41 (27.5%)subjects (Table-V). Nearly all diabetic patients (98%) with OA has high WC (p = 0.04). Female diabetic subjects having high WC were more frequent to have OA (n= 42, 31%) than male subjects (n= 9, 16%). Similarly, female diabetic patients with BMI ≥25 were more likely to have OA (32.6%) than male diabetic cohort (18.3%) in the same BMI category (Table-VI).

**Table-I:** Demographics of diabetic and non diabetic groups.

*Basic information*	*Diabetic n(%)*	*Non-diabeticn(%)*	*Overall n(%)*
Age (Mean±SD) years	50.70±10.20	49.52±10.57	50.12±10.9
n	210	203	413
Gender			
Male	72 (34.3)	71 (35.0)	143(34.6)
Female	138 (65.7)	132 (65.0)	270(65.4)
N			413
Diabetes Duration (Mean±SD)years	6.27±5.74	--	6.27±5.74
n	210	0	210

**Table-II:** Correlation of kOA with diabetes mellitus.

*Study Group*				

*Osteoarthritis*	*Diabetic n(%)*	*Control n(%)*	*Overall n(%)*	*p-value*
+ve	52(24.8)	54(26.6)	106(25.7)	0.669
-ve	158(75.2)	149(73.4)	307(74.3)	
n	210	203	413	

**Table-III:** Odd ratio of Knee OA according to different variables in diabetic group.

*Variables*	*Odd Ratio*	*95% CI*	*n*
BMI	1.043	0.984- 1.106	210
WC	1.016	0.988- 1.045	210
Age	1.027	0.995- 1.059	210
Duration of Diabetes	1.058	1.005- 1.114	
*Gender*			210
Male	1	1.269- 5.800	210
Female	2.712		
*BMI Category*			210
Normal	1	--	
Over weight	0.825	0.206- 3.313	
Obese	1.645	0.631- 4.287	
*BMI Tertiles*			210
tertile 1	1	--	
tertile 2	1.234	0.556- 2.740	
tertile 3	1.721	0.801- 3.695	
*WC Tertiles*			210
tertile 1	1	--	
tertile 2	1.854	0.848- 4.052	

**Table-IV:** Odd ratio of KOA(+ve) in relation to age, gender and diabetes mellitus (n413).

*Variables*	*Odd Ratio*	*95% CI*	*n*
Age	1.039	1.017- 1.062	413
*Gender*			413
Male	1	--	
Female	2.163	1.299- 3.600	
*Study Group*			413
Non-diabetes	1	--	
Diabates	0.908	0.584- 1.412	

**Table-V:** Frequencies of KOA in normal, overweight and obese category of BMI.

*Gender*	*BMI*	*Osteoarthritis*		*Total*

		*+VE*	*-VE*
Female	18.5-22.9	6 17		23
	23-24.9	4 13		17
	≥25	32 66		98
		42(20%) 96(46%)		138
Male	18.5-22.9	1 11		12
	23-24.9	0 11		11
	≥25	9 40		49
		10(5%) 62 (29%)		72

**Table-VI:** Frequency of KOA in normal and high category of WC by Gender.

	*Waist Circumference Category (Male)*			

*Osteoarthritis*	*Normal n(%)*	*High n(%)*	*Overall n(%)*	*p-value*
+ve	1(6.3)	9(16.1)	10(13.9)	0.44
-ve	15(93.8)	47(83.9)	62(86.1)	
n	16	56	72	

	*Waist Circumference Category (Female)*			

*Osteoarthritis*	*Normal n(%)*	*High n(%)*	*Overall n(%)*	*p-value*

+ve	0(0)	42(31.1)	42(30.4)	0.553
-ve	3(100)	93(68.9)	96(69.6)	
n	3	135	138	

	*Waist Circumference Category (Overall)*			

*Osteoarthritis*	*Normal n(%)*	*High n(%)*	*Overall n(%)*	*p-value*

+ve	1(5.3)	51(26.7)	52(24.8)	0.049
-ve	18(94.7)	140(73.3)	158(75.2)	
n	19	191	210	

## DISCUSSION

Hip and knee OA is 11th highest contributor and one of the leading causes of global disability. The global age-standardised prevalence of knee OA is 3.8% higher in females.^13^ The association between obesity and kOA, and specifically the role of obesity as a risk factor for kOA has been well documented.^14^

Felson et al not only showed a causative relationship between obesity and kOA but also showed that the effect was stronger in women than in men, in the same study he proved that weight loss significantly reduced the risk for symptomatic kOA in women.^15^

Results from a meta-analysis found out that among the other risk factors, obesity (pooled OR 2.63), female gender (pooled OR 1.84) and older age were consistently associated with knee OA a finding very similar in our study.^16^ Furthermore, it has been established that weight loss for obese patients with kOA results in clinical improvement, as far as pain reduction and improved function is concerned, however the exact mechanism linking obesity and osteoarthritis is complex.^17^

Obesity has not only been elaborated as a causative factor for kOA, but it has been observed that in obese subjects, the results of total knee arthroplasty may be compromised by postoperative complications, particularly infection.^18^

Visser et al looked into the relationship between fat mass (FM) and skeletal muscle mass (SMM) with OA. They showed that clinical or structural OA was present in 25% and 14% in females with high FM and fat percentages were positively associated with clinical kOA in both men and women. The FM/SMM ratio was also positively associated with clinical OA.^19^

The magnitude of the association varies by sex as a 5-unit increase in BMI in females was found to be associated with a 35% increased risk of kOA^8^. Similarly, the relative risk of kOA is 8.1 for BMI, and 6.7 for waist circumference (WC) found in another study by Lohmander LS.^20^

There are some other exceptional reports which point to a relatively insignificant association of obesity with kOA.^21^ Han et al in a Korean cohort, found out that among the parameters of Metabolic Syndrome, only high waist circumference (WC) was significantly associated with an increased prevalence of kOA and in female subjects alone.^22^-^23^

While the association of obesity with OA and kOA has been well documented in the literature’^23^ the relationship between diabetes mellitus with kOA has been inconsistent, our study has also shown similar results with no major difference in frequencies of knee OA in diabetics or non-diabetics.

Schett G, Kleyer A^24^ concluded in their study that Type 2 diabetes predicts the development of severe OA independent of age and BMI, in our study however, this has been proven wrong as increasing age, female gender and obesity are all contributors in KOA whereas diabetes has no significant association. The results of our study however cannot be compared with international data due to the varied geographical differences, in addition there are other confounding factors like average BMI in the population, occupation and life style which are not the same therefore, the difference in opinion.

The duration of diabetes and age has shown a positive correlation with the frequency of kOA as in many other studies, however, the two factors may synergize in some cases as the older the age of a diabetic patient, the greater would be the disease duration too and hence the more chances of having kOA.

The primary objective was to determine if BMI and/or WC has significant correlation with KOA so they can be considered as independent predictor for kOA. The results of this study supports the existing published data in this respect which emphasizes the importance of weight control in prevention as well as progression of KOA. Irrespective of the results, in subjects having diabetes mellitus 70% fall into the category of obese; similarly 80% have a high WC which represents the altitude of problem regarding awareness of a healthy lifestyle and weight consciousness.

Female subjects in our cohort with a high WC have shown a greater percentage of osteoarthritis 31% than with normal or with males in the same category which represents only 16%. Similarly, females in obese category has more OA 32% than males in the same category 9%, however this may be due to the wide range of BMI after 25.

This finding however can be justified by the fact that females carry much of their fat in their lower extremities, whereas men has adipose tissue at the abdominal level and is consistent with the results of several other studies.^25^,^26^

Our study emphasis the importance of WC as a definite predictive anthropometric measure in addition to BMI for the presence of kOA, as the role of adipocytes has already been discussed. Therefore, lifestyle modification including non weight bearing exercises and weight reduction should be the first and the foremost non pharmacological approach which would definitely benefit these patients. The study should also be replicated in rural population to observe other possible effects like heavy physical work on knee OA where people are exposed to heavy work to earn a living.

### Strengths and Limitations

A large homogenous sample of consecutive patients were enrolled in this study which limits the selection bias. The BMI and WC were measured by the same nursing staff which limits variability in data.

The limitation remains that BMI and WC should have been measured in nondiabetic subjects and compared with the diabetic cohort, but we have compared our data with other studies regarding the affect of BMI and WC on OA. This study is important since no published data is available in this region showing the relationship of WC to predict knee OA.
